# Efficacy of renal replacement therapy in critically ill patients: a propensity analysis

**DOI:** 10.1186/cc11905

**Published:** 2012-12-19

**Authors:** Christophe Clec'h, Michaël Darmon, Alexandre Lautrette, Frank Chemouni, Elie Azoulay, Carole Schwebel, Anne-Sylvie Dumenil, Maïté Garrouste-Orgeas, Dany Goldgran-Toledano, Yves Cohen, Jean-François Timsit

**Affiliations:** 1 Medical-surgical ICU, Avicenne Teaching Hospital, 125 route de Stalingrad, Bobigny, 93000, France; 2 Albert Bonniot Institute, INSERM U823, University Grenoble 1, rond-point de la Chantourne, La Tronche, 38700, France; 3 Medical ICU, Saint-Etienne Teaching Hospital, avenue Albert Raimond, Saint-Priest-en-Jarez, 42270, France; 4 Medical ICU, Gabriel Montpied Teaching Hospital, 58 boulevard Montalembert, Clermont-Ferrand, 63003, France; 5 Saint-Louis Teaching Hospital, 1 avenue Claude-Vellefaux, Paris, 75010, France; 6 Medical ICU, Albert Michallon Teaching Hospital, boulevard de la Chantourne, La Tronche, 38700, France; 7 Medical-surgical ICU, Antoine Béclère Teaching Hospital, 157 rue de la Porte de Trivaux, Clamart, 92140, France; 8 Medical-surgical ICU, Saint-Joseph Hospital, 185 rue Raymond Losserand, Paris, 75014, France; 9 Medical-surgical ICU, Gonesse Hospital, 25 rue Bernard Février, Gonesse, 95500, France

## Abstract

**Introduction:**

Although renal replacement therapy (RRT) is a common procedure in critically ill patients with acute kidney injury (AKI), its efficacy remains uncertain. Patients who receive RRT usually have higher mortality rates than those who do not. However, many differences exist in severity patterns between patients with and those without RRT and available results are further confounded by treatment selection bias since no consensus on indications for RRT has been reached so far. Our aim was to account for these biases to accurately assess RRT efficacy, with special attention to RRT timing.

**Methods:**

We performed a propensity analysis using data of the French longitudinal prospective multicenter Outcomerea database. Two propensity scores for RRT were built to match patients who received RRT to controls who did not despite having a close probability of receiving the procedure. AKI was defined according to RIFLE criteria. The association between RRT and hospital mortality was examined through multivariate conditional logistic regression analyses to control for residual confounding. Sensitivity analyses were conducted to examine the impact of RRT timing.

**Results:**

Among the 2846 study patients, 545 (19%) received RRT. Crude mortality rates were higher in patients with than in those without RRT (38% vs 17.5%, *P *< 0.001). After matching and adjustment, RRT was not associated with a reduced hospital mortality. The two propensity models yielded concordant results.

**Conclusions:**

In our study population, RRT failed to reduce hospital mortality. This result emphasizes the need for randomized studies comparing RRT to conservative management in selected ICU patients, with special focus on RRT timing.

## Introduction

Acute kidney injury (AKI) significantly contributes to the morbidity and the mortality of critically ill patients through metabolic derangements, fluid overload and harmful effects of these disturbances on other failing organs. Renal replacement therapy (RRT), although not achieving the same level of homeostasis as a normally functioning kidney, helps limit the consequences of AKI and allows adequate administration of fluids and nutritional support. However, its benefits (aside from life-threatening complications, such as severe hyperkalemia, pulmonary edema, and intractable acidosis) in critically ill patients with AKI remain unclear.

Available data are derived from uncontrolled studies, which all showed higher mortality rates among populations treated with RRT [1-5]. Due to their design, however, confounders and biases may have limited their accuracy. Particularly, treatment selection bias [6] may have confounded the results. This kind of bias occurs when no agreed-upon indications exist for a given treatment or procedure, which is the case for RRT despite the recent publication of recommendations for the prevention and management of AKI in the intensive care unit (ICU) [7]. Since there are no clear guidelines about whether and when RRT should be started, patients' characteristics, in-ICU events, and other aspects of ICU care, which may also affect outcomes, may confound the analysis of RRT efficacy, leading to inconclusive results. The propensity score technique described by Rosenbaum and Rubin is a powerful method to control for treatment selection bias [8,9]. The aim of this study was to use the propensity technique to estimate the association of RRT with in-hospital mortality in ICU patients with AKI.

## Materials and methods

### Study design and data source

We conducted an observational study in a multiple-center database (OUTCOMEREA) from January 1997 to June 2009. Methods of data collection and quality of the database have been described in details elsewhere [10]. Briefly, a large set of data on a random sample of patients older than 16 years with ICU stays longer than 24 h was prospectively collected by the senior physicians of the participating ICUs and entered into the database each year. The quality control procedure involved multiple automatic checking of internal consistency and biennial audits.

### Ethics approval

In accordance with French law, the OUTCOMEREA database was declared to the Commission Nationale de l'Informatique et des Libertés. The study was approved by the ethics committee of Clermont-Ferrand, France. Since the study did not modify patients' management and data were processed anonymously, the need for informed consent was waived.

### Study population and definitions

All patients in the database were eligible. Exclusion criteria were: chronic kidney disease (CKD) (with or without complete loss of kidney function), pre-renal cause of renal dysfunction (that is rapidly reversible functional renal failure), multiple ICU stays, decision to withhold or withdraw life-sustaining treatments, and renal replacement therapy for extra-renal indications (such as, intoxications or cardiogenic shock). CKD was defined either according to the Acute Physiology and Chronic Health Evaluation (APACHE) II definition or a specific code in the database when not requiring dialysis. Pre-renal cause of renal dysfunction was also identified through a specific code in the database. The reason for excluding these patients was that their prognosis may be different from that of patients with prior normal renal function who present a non-rapidly reversible cause of AKI. Patients with multiple ICU stays, or with a decision to withhold or withdraw life-sustaining treatments, were also excluded to avoid confusion in the assessment of hospital mortality.

Among the remaining patients, those in whom AKI occurred were analyzed. AKI was defined according to the RIFLE (Risk, Injury, Failure, Loss and End-stage renal failure) criteria [11], and patients were classified according to the maximum RIFLE class (Risk, Injury or Failure) reached during their ICU stay. The maximum RIFLE class was determined before RRT initiation in patients who received RRT and whenever during the ICU stay in patients who did not. Since the 6- and 12-h urine outputs were not recorded in the database, we used the glomerular filtration rate (GFR) only. The GFR criteria were determined according to changes in serum creatinine from baseline values. As AKI may be present on ICU admission in a high proportion of patients, we chose to assess baseline creatinine values using the Modification of Diet in Renal Disease (MDRD) equation. As recommended by the Acute Dialysis Quality Initiative Group, a normal GFR of 75 ml/min/1.73 m^2 ^before ICU admission was assumed [11].

RRT consisted of intermittent hemodialysis or continuous veno-venous hemofiltration/hemodiafiltration. All participating centers were able to provide both techniques of RRT. The decision to start RRT was left at the discretion of the attending ICU physicians.

### Data collection

The following data were recorded:

• baseline characteristics on ICU admission: age, sex, McCabe class (class 1, no fatal underlying disease; class 2, underlying disease fatal within five years; class 3, underlying disease fatal within one year), Simplified Acute Physiology Score (SAPS) II, comorbidities assessed according to the APACHE II definitions, transfer from ward (defined as a stay in an acute-bed ward ≥24 hrs immediately before ICU admission), and admission category (medical, scheduled surgery, or unscheduled surgery),

• during the ICU stay: daily biological parameters (blood urea nitrogen, serum creatinine, kaliemia), daily urine output, daily weight, time from admission to maximum RIFLE class, time to RRT, daily Sequential Organ Failure Assessment (SOFA) score, and modified SOFA score (mSOFA, SOFA - specific renal component),

• on ICU discharge: renal status (recovery or need for prolonged renal support), and length of ICU stay, and,

• on hospital discharge: vital status.

### Endpoints

The primary endpoint was hospital mortality.

The secondary endpoints were the length of ICU stay, and renal status on ICU discharge.

### Propensity technique

Since RRT was not randomly assigned in our study population, treatment selection bias was accounted for by using the propensity technique. When building a propensity score, the main risk is the omission of an important variable in the propensity regression. Thus, we fitted and compared two different models to strengthen our analysis. As recommended, propensity scores were determined through multivariate logistic regression [12], in which RRT was the dependent variable. Independent variables were related to the probability of receiving the treatment and also outcome in order to reduce both the bias and the variance in the estimation of treatment effect [13,14]. Independent variables introduced in model 1 were: rising creatinine reflected by maximum RIFLE class, oliguria reflected by the 24-h urine output on reaching maximum RIFLE class, and SAPS II score. Independent variables introduced in model 2 were: blood urea nitrogen, serum creatinine and kaliemia measured on reaching maximum RIFLE class (that is, before RRT was started), fluid accumulation (reflected by the difference between patients' weight recorded on reaching maximum RIFLE class and that recorded on ICU admission), and SAPS II score.

Using an algorithm [15], we matched patients who received RRT during their ICU stay to other AKI patients who did not on the basis of each of the two propensity scores that we built (model 1 and model 2). Specifically, we sought to match each patient with RRT up to three controls who had the closest propensity score (within 0.05 on a scale of 0 to 1).

Besides, patients were also matched on center and period of admission to account for possible inconsistent institutional practices or changes in RRT practices over time. Age (+/- 5 years) was the final matching criterion. The adequacy of the propensity scores in controlling for treatment selection bias was demonstrated by testing for differences between matched patients in biological parameters likely to trigger RRT on reaching maximum RIFLE class.

The goodness of fit and the discrimination of the two logistic regression models used to derive a propensity score for RRT were evaluated by the Hosmer-Lemeshow (HL) test, and the c statistic (area under the receiver operating characteristics curve), respectively.

### Statistical analyses

Results are expressed as numerical values and percentages for categorical variables, and as means and standard deviations (SD) or medians and quartiles [Q1-Q3] for continuous variables.

In the whole cohort, comparisons of patients with and those without RRT were based on chi-square tests for categorical data, and on Student's t-test or Wilcoxon's test for continuous data, as appropriate.

Comparisons between matched patients were based on univariate conditional logistic regression. Multivariate conditional logistic regression analysis was used to examine the association between RRT and subsequent hospital mortality, adjusting for variables potentially related to mortality that were not considered in the propensity regression (namely baseline characteristics that had a *P *value < 0.1 in univariate analysis, and the modified SOFA score (SOFA score - specific renal component) computed on the day maximum RIFLE class was reached).

Sensitivity analyses were performed to test whether any delay in RRT initiation could affect patients' prognosis. For that purpose, the timing of RRT was divided into three classes (less than 24 h, between 24 and 48 h, greater than 48 h after reaching maximum RIFLE class).

Since the use of the MDRD equation to estimate baseline creatinine values has not been validated in ICU patients, we also performed sensitivity analyses that included only patients with a normal serum creatinine value measured on ICU admission.

Wald χ2 tests were used to determine the significance of each variable. Adjusted odds ratios (ORs) and 95% confidence intervals (CIs) were calculated for each parameter estimate.

Analyses were computed using the SAS 9.1 software package (SAS Institute, Cary, NC, USA).

## Results

### Study patients

Over the study period, 10,911 patients with a single ICU stay were screened, of whom 2,272 were excluded for the following reasons: decision to withhold or withdraw life-sustaining treatments (*n *= 1,378, 12.6%), history of chronic kidney disease (*n *= 672, 6.2%), functional renal failure (*n *= 176, 1.6%), and RRT for extra-renal indications (*n *= 46, 0.4%).

Among the remaining 8,639 patients, 2,846 (32.9%) had AKI (1,025 (36%) R class patients, 830 (29.2%) I class patients, and 991 (34.8%) F class patients).

RRT was initiated in 545 (19.1%) AKI patients (41 (7.5%) R class patients, 110 (20.2%) I class patients, and 394 (72.3%) F class patients).

Patients who received RRT were younger, had higher severity scores, were more likely to be transferred from ward, and presented more comorbidities than patients who did not receive RRT (Table 1). Differences between patients with and without RRT according to the maximum RIFLE class reached during the ICU stay are shown in Additional files 1, 2, and 3.

**Table 1:** Baseline characteristics of acute kidney injury (AKI) patients with and without renal replacement therapy (RRT).

Variable	Patients with RRT (*N *= 545)	Patients without RRT (*N *= 2301)	*P *value
Age, mean (SD)	61.3 (16.6)	67.6 (15.5)	< 0.0001
Males, no. (%)	363 (66.6)	1309 (59.9)	< 0.0001
SAPS II score, mean (SD)	56.8 (19.2)	48.6 (19.8)	< 0.0001
APACHE II score, mean (SD)	21.4 (7.0)	19.6 (7.1)	< 0.0001
Transfer from ward, no. (%)	291 (53.4)	1072 (46.6)	0.004
McCabe, no. (%)			
1	314 (57.6)	1352 (58.8)	
2	188 (34.5)	771 (33.5)	0.88
3	43 (7.9)	178 (7.7)	
Admission category, no. (%)			
Medical	388 (71.2)	1655 (71.9)	
Scheduled surgery	52 (9.5)	259 (11.3)	0.25
Unscheduled surgery	105 (19.3)	387 (16.8)	
Chronic coexisting conditions, no. (%)			
Cardiac disease	89 (16.3)	420 (18.3)	0.29
Respiratory disease	55 (10.1)	311 (13.5)	0.03
Liver disease	50 (9.2)	128 (5.6)	0.002
Immunodeficiency	104 (19.1)	336 (14.6)	0.01
Uncomplicated diabetes mellitus	63 (11.6)	257 (11.2)	0.79
Complicated diabetes mellitus	30 (5.5)	118 (5.1)	0.72

SAPS, Simplified Acute Physiology Score; APACHE, Acute Physiology and Chronic Health Evaluation.

### Dynamics of AKI and timing of renal replacement therapy

AKI occurred early in the course of ICU stay. Three-quarters of the patients reached their maximum RIFLE within three days after ICU admission.

When a decision of RRT was made, RRT was started less than 48 h after reaching maximum RIFLE class in 479/545 (87.9%) patients. Continuous veno-venous hemofiltration/hemodiafiltration and intermittent hemodialysis were used as initial RRT modality in 345 (63.3%) patients and 200 (36.7%) patients, respectively.

Details on timings of AKI and RRT for each RIFLE class are shown in Tables 2 and 3.

**Table 2:** Timing of acute kidney injury (AKI).

	All patients	R class patients	I class patients	F class patients
Time to AKI onset*	2 [1-2]	1 [1-2]	2 [1-2]	1 [1-2]
Time to maximum RIFLE class*	2 [1-3]	1 [1-2]	2 [1-3]	2 [1-3]

Results are expressed as medians (in days) and [interquartile range]. *From ICU admission (day 0).

**Table 3:** Timing of renal replacement therapy initiation.

	All patients	R class patients	I class patients	F class patients
Time from AKI onset	1 [0-3]	0 [0-1]	1 [0-2]	1 [0-3]
Time from maximum RIFLE class	0 [0-1]	0 [0-1]	0 [0-1]	0 [0-1]

Results are expressed as medians (in days) and [interquartile range]. AKI, acute kidney injury.

Differences in parameters (measured on reaching maximum RIFLE class) likely to trigger RRT between patients who actually received RRT and those who did not are presented in Table 4. Patients with RRT had higher blood urea nitrogen, serum creatinine and kaliemia but their pH values were not significantly lower.

**Table 4:** Differences in parameters likely to trigger renal replacement therapy (RRT) on reaching maximum RIFLE class between patients with and without RRT (whole cohort).

	Patients with RRT *N *= 545	Patients without RRT *N *= 2301	*P *value
Urea (mmol/L)	20 [14-28]	14 [10-20]	< 0.001
Creatinine (mmol/L)	305 [231-408]	178 [143-247]	< 0.001
Potassium (mmol/L)	4.3 [3.8-5.1]	4.2 [3.7-4.7]	< 0.001
pH	7.34 [7.24-7.43]	7.36 [7.27-7.44]	0.4
mSOFA	7 [4-10]	5 [2-7]	< 0.001
SAPS II	49 [38-62]	41 [32-53]	< 0.001
Urine output (L)	0.4 [0.1-1.1]	1.3 [0.6-2.3]	< 0.001
Fluid accumulation (L)	4 [2-8]	2 [0-4]	< 0.001

Results are expressed as medians and [interquartile range]. SOFA, Sequential Organ Failure Assessment; mSOFA, SOFA - specific renal component; SAPS, Simplified Acute Physiology Score.

### Matching on the propensity scores

The two propensity models showed satisfying goodness of fit and discrimination (*P *values for the HL test: 0.39 and 0.52, c statistics: 0.80 and 0.78, in models 1 and 2, respectively). The percentage of matched patients was high despite numerous and strict matching criteria. In model 1, 383/545 (70%) patients who received RRT could be matched to 726 controls who did not receive RRT. In model 2, 376/545 (69%) RRT patients could be matched to 754 controls. In both models, there were no differences between patients with and those without RRT in biological parameters likely to trigger RRT on reaching maximum RIFLE class (Table 5), thus confirming the ability of the propensity scores to control for treatment selection bias. However, there remained differences in SAPS II, mSOFA, urine output and fluid accumulation that were thus adjusted for (Table 5).

**Table 5:** Differences in parameters likely to trigger renal replacement therapy (RRT) on reaching maximum RIFLE class between patients with and without RRT (matched patients).

	Patients with RRT	Patients without RRT	*P *value
**Model 1**	*N *= 383	*N *= 726	
Urea (mmol/L)	15 [10-23]	15 [11-24]	0.3
Creatinine (mmol/L)	217 [158-281]	214 [158-271]	0.4
Potassium (mmol/L)	4.1 [3.6-4.7]	4.1 [3.6-4.6]	0.9
pH	7.34 [7.24-7.44]	7.36 [7.28-7.44]	0.5
mSOFA	7 [5-9]	5 [3-8]	< 0.001
SAPS II	50 [38-62]	43 [34-56]	< 0.01
Urine output (L)	0.6 [0.2-1.3]	1.4 [0.7-2.5]	< 0.001
Fluid accumulation (L)	4 [2-8]	2 [0-6]	< 0.01
			
**Model 2**	*N *= 376	*N *= 754	
Urea (mmol/L)	16 [10-23]	14 [10-22]	0.9
Creatinine (mmol/L)	217 [158-286]	203 [144-265]	0.12
Potassium (mmol/L)	4.1 [3.6-4.8]	4.1 [3.6-4.6]	0.4
pH	7.33 [7.24-7.43]	7.36 [7.27-7.45]	0.3
mSOFA	7 [5-10]	5 [3-8]	< 0.001
SAPS II	50 [39-63]	42 [34-54]	< 0.001
Urine output (L)	0.6 [0.2-1.2]	1.2 [0.5-2.2]	< 0.001
Fluid accumulation (L)	4 [2-8]	2 [1-6]	< 0.03

Results are expressed as medians and [interquartile range]. SOFA, Sequential Organ Failure Assessment; mSOFA, SOFA - specific renal component; SAPS, Simplified Acute Physiology Score.

### Impact of renal replacement therapy

RRT resulted in longer lengths of ICU stay after reaching maximum RIFLE class (see Additional files 4 and 5) but did not reduce mortality. Crude hospital mortality rates of patients with and without RRT were 45.1% and 23.4%, respectively (*P *< 0.001). Among patients who received RRT, 92 of the 338 survivors (27.2%) still needed renal support on ICU discharge.

After matching on the propensity scores, patients who received RRT still had higher mortality rates than their respective controls (model 1: 38.9% vs 22.2%, *P *< 0.001; model 2: 38% vs 18.3%, *P *< 0.001), in univariate analysis. After adjustment on confounding variables, RRT was not associated with a reduced hospital mortality, whatever its timing (Table 6). Additional files 6 and 7 show details according to the maximum RIFLE class reached during the ICU stay.

**Table 6:** Association of renal replacement therapy (RRT) with hospital mortality in multivariate conditional logistic regression (matched patients) according to timing of RRT.

	OR	95% CI	*P *value
**Model 1**			
All RRT (whatever the timing)	1.30	0.96-1.78	0.09
Immediate RRT*	1.43	0.91-2.22	0.12
Early RRT**	0.99	0.45-2.15	0.92
Delayed RRT***	2.37	1.04-5.40	0.04
			
**Model 2**			-
All RRT (whatever the timing)	1.41	1.02-1.94	0.04
Immediate RRT*	1.14	0.71-1.81	0.59
Early RRT**	2.31	0.96-5.67	0.06
Delayed RRT***	2.29	1.05-4.99	0.04

OR, odds ratio; CI, confidence interval. *Initiated within 24 h after reaching maximum RIFLE class; **initiated between 24 and 48 h after reaching maximum RIFLE class; ***initiated more than 48 h after reaching maximum RIFLE class.

The sensitivity analyses that included only patients with a normal serum creatinine value measured on ICU admission yielded similar results as the full analysis (Additional file 8).

## Discussion

While the impact of RRT modalities has been widely investigated through randomized controlled trials [16-21], the overall efficacy of RRT remains uncertain. Actually, there is no real head-to-head comparison of AKI patients with and without RRT in the current literature. Mortality rates are usually higher in patients with than in those without RRT [1-5]. However, no definitive conclusions can be can be drawn from these data due to the absence of clear indications for RRT and the many differences in severity patterns between patients who receive RRT and those who do not. In other words, treatment selection bias and patients' underlying severity are major confounders making the assessment of RRT efficacy challenging.

Our study brings a new insight in the field. By using the propensity technique, we were able to compare hospital mortality rates in matched patients with and without RRT, having a close probability of receiving RRT (somewhat as though RRT had been 'randomly assigned'). Moreover, since the SAPS II score was included in the propensity regressions, matched patients with and without RRT had also a similar predicted hospital mortality. Consequently, the risk of biased assessment of the association between RRT and hospital mortality was minimized.

Like in the interesting study of Elseviers *et al*. [22] that reported an increased risk of death for RRT compared to conservative treatment in ICU patients after extensive adjustment on disease severity, we failed to demonstrate any beneficial effect of RRT. While it cannot be totally run out that RRT *per se *is potentially harmful (hemodynamic instability, central venous catheter-related blood stream infections, inflammation and coagulation disorders, which are common complications of RRT, may well have outweighed its metabolic benefits), these results emphasize the need for a critical reappraisal of current RRT practices and definitions of AKI. Particularly, it must be kept in mind that timing of RRT initiation is undoubtedly a key issue. In this regard, a plausible explanation for our findings is that RRT was in fact initiated too late. Actually, patients were classified according to the glomerular filtration rate (GFR) criteria of RIFLE whereas increases in serum creatinine often lag behind the true reduction in GFR. Thus, although RRT was in place within 24 h after reaching maximum RIFLE class in the vast majority of patients, it might well have been initiated at a more advanced stage of renal dysfunction than clinically appreciated. So, our results do not imply, as one may believe at first sight, that RRT should be abandoned. Rather, the key message could be: 'initiate RRT as early as possible'. That patients who received RRT had more coexisting organ failures on reaching maximum RIFLE class than their matched controls lends support to this hypothesis of delayed AKI diagnosis and RRT. Since initiation of RRT when multiple organ failures are present probably limits its ability to improve patients' outcomes, the utilization of highly sensitive and early diagnostic biomarkers such as cystatin C or neutrophil gelatinase-associated lipocalin (instead of serum creatinine) as triggers for RRT is worth considering for future investigations in the ICU [23-30].

Despite the use of an original statistical approach minimizing the risk of bias, our study has potential limitations that merit consideration.

First, residual confounding cannot be totally excluded because of the observational design. However, by applying the propensity technique and matching on age, and center and period of admission, we dealt with confounding more extensively than in prior reports. Besides, that the two propensity models yielded similar results made the hypothesis of having omitted an important confounding variable unlikely.

Second, we encountered the same problem as others [31,32]: the 6- and 12-h urine outputs were not recorded in our database. Therefore, patients were classified according to the GFR criteria only. Patients classified according the GFR criteria seem to be more severely ill and have slightly higher mortality rates than their counterparts classified according to the urine output criteria [33,34]. Having considered both criteria may have resulted in a different estimation of RRT efficacy. Yet, urine output does not differentiate functional (pre-renal) AKI from organic AKI and new serum or urine biomarkers are probably much more reliable for the early diagnosis of AKI.

Third, the MDRD equation used to estimate baseline creatinine values has not been validated in ICU patients. Nevertheless, the sensitivity analysis including only data from patients with a normal serum creatinine value on ICU admission yielded similar results as the full analysis, showing that the use of the MDRD equation did not bias the results.

Fourth, the use of the MDRD equation to estimate baseline creatinine values refrains from precisely establishing AKI onset (that is, patients with an apparent early-onset AKI may in fact have developed AKI for several days before ICU admission). This could be problematic in that the prognosis of early AKI may differ from that of late AKI. That results of the sensitivity analysis, including only data from patients with a normal serum creatinine value on ICU admission, yielded similar results as the full analysis runs counter to the hypothesis of differential prognosis and impact of RRT between early and late AKI. However, this issue needs further evaluation.

Fifth, it might be argued that RRT initiation may have prevented R or I class patients from reaching a higher RIFLE class (thus leading to an underestimation of their degree of renal dysfunction, and subsequent comparison of RRT patients with non-RRT patients having a more severe renal dysfunction). Yet, this limit, which is inherent to the RIFLE classification, does not apply to F class patients. Since odds ratios of mortality associated with RRT in the whole population were similar as those in the F class patients, it is very unlikely that results were flawed by a potential misclassification bias induced by an underestimation of renal dysfunction in RRT patients.

Sixth, the prognostic impact of the dose and initial modality of RRT was not assessed. It must be emphasized, however, that all randomized controlled trials conducted so far have showed equivalence between high and low doses, and continuous and intermittent RRT [16-21].

Finally, data on the long-term impact of AKI and RRT were not recorded in the database, and concomitant measures likely to prevent or positively influence the course of renal dysfunction (optimization of hemodynamics and renal perfusion, avoidance of nephrotoxic drugs) were not analyzed. These issues deserve future prospective evaluations.

## Conclusions

Together with those of Elseviers *et al*. [22], our findings raise concern about the actual efficacy of RRT. Of course, these results must be cautiously interpreted since the assessment of RRT efficacy through observational data is very challenging. However, they emphasize the need for a critical reappraisal of current RRT practices. Large randomized controlled trials comparing RRT to conservative management in selected ICU patients with AKI, and focusing on RRT timing, are urgently warranted to provide definite conclusions.

## Key messages

• Aside from life-threatening conditions, evidence supporting the use of renal replacement therapy (RRT) in critically ill patients with acute kidney injury (AKI) is lacking. Currently available data on RRT efficacy exclusively stem from observational studies, whose results may have been confounded by treatment selection bias and differences in patients' severity.

• In this study, we extensively dealt with confounding by using the propensity score technique and multivariate regression models to provide an as accurate as possible estimation of RRT efficacy.

• RRT was not associated with decreased mortality and even seemed to impair patients' outcome when initiated too late.

• These results emphasize the need for further randomized studies comparing RRT to conservative management in selected ICU patients, with special focus on RRT timing.

## Abbreviations

AKI: acute kidney injury; APACHE: Acute Physiology and Chronic Health Evaluation; CKD: chronic kidney disease; CI: confidence interval; GFR: glomerular filtration rate; ICU: intensive care unit; MDRD: Modification of Diet in Renal Disease; OR: odds ratio; RIFLE: Risk: Injury: Failure: Loss and End-stage renal failure; RRT: renal replacement therapy; SAPS: Simplified Acute Physiology Score; SOFA: Sequential Organ Failure Assessment.

## Competing interests

The authors declare that they have no competing interests.

## Authors' contributions

CC designed the study and wrote the manuscript; CC and JFT performed the statistical analyses; MD, AL, FC, EA, CS, ASD, MGO, DGT, and YC participated in the collection of data and critically revised the manuscript for important intellectual content. All authors read and approved the final manuscript.

## Supplementary Material

Additional file 1 **Baseline characteristics of RIFLE R class patients with and without renal replacement therapy (RRT)**. Click here for file

Additional file 2 **Baseline characteristics of RIFLE I class patients with and without renal replacement therapy (RRT)**. Click here for file

Additional file 3 **Baseline characteristics of RIFLE F class patients with and without renal replacement therapy (RRT)**. Click here for file

Additional file 4 **Lengths of ICU stay after reaching maximum RIFLE class in patients with and without renal replacement therapy (RRT)**. Click here for file

Additional file 5 **Lengths of ICU stay after reaching maximum RIFLE class in nonsurvivors with and without renal replacement therapy (RRT)**. Click here for file

Additional file 6 **Association of renal replacement therapy (RRT) with hospital mortality in multivariate conditional logistic regression according to timing of RRT and maximum RIFLE class reached during the ICU stay (model 1)**. Click here for file

Additional file 7 **Association of renal replacement therapy (RRT) with hospital mortality in multivariate conditional logistic regression according to timing of RRT and maximum RIFLE class reached during the ICU stay (model 2)**. Click here for file

Additional file 8 **Association of renal replacement therapy (RRT) with hospital mortality in multivariate conditional logistic regression (matched patients) according to timing of RRT: results of sensitivity analyses including only patients with a normal serum creatinine value measured on ICU admission**. Click here for file

## References

[B1] Brivet FG Kleinknecht DJ Loirat P Landais PJ Acute renal failure in intensive care units--causes, outcome, and prognostic factors of hospital mortality; a prospective, multicenter study. French Study Group on Acute Renal Failure Crit Care Med 1996 16 192 198 10.1097/00003246-199602000-00003 8605788

[B2] Liano F Junco E Pascual J Madero R Verde E The spectrum of acute renal failure in the intensive care unit compared with that seen in other settings. The Madrid Acute Renal Failure Study Group Kidney Int Suppl 1998 16 S16 24 9580541

[B3] Mehta RL Pascual MT Gruta CG Zhuang S Chertow GM Refining predictive models in critically ill patients with acute renal failure J Am Soc Nephrol 2002 16 1350 1357 10.1097/01.ASN.0000014692.19351.52 11961023

[B4] Mehta RL Pascual MT Soroko S Savage BR Himmelfarb J Ikizler TA Paganini EP Chertow GM Spectrum of acute renal failure in the intensive care unit: the PICARD experience Kidney Int 2004 16 1613 1621 10.1111/j.1523-1755.2004.00927.x 15458458

[B5] Metnitz PG Krenn CG Steltzer H Lang T Ploder J Lenz K Le Gall JR Druml W Effect of acute renal failure requiring renal replacement therapy on outcome in critically ill patients Crit Care Med 2002 16 2051 2058 10.1097/00003246-200209000-00016 12352040

[B6] Greenland S Neutra R Control of confounding in the assessment of medical technology Int J Epidemiol 1980 16 361 367 10.1093/ije/9.4.361 7203778

[B7] Brochard L Abroug F Brenner M Broccard AF Danner RL Ferrer M Laghi F Magder S Papazian L Pelosi P Polderman KH An Official ATS/ERS/ESICM/SCCM/SRLF Statement: Prevention and Management of Acute Renal Failure in the ICU Patient: an international consensus conference in intensive care medicine Am J Respir Crit Care Med 2010 16 1128 1155 10.1164/rccm.200711-1664ST 20460549

[B8] Rosenbaum PR Rubin DB The central role of the propensity score in observational studies for causal effects Biometrika 1983 16 41 55 10.1093/biomet/70.1.41

[B9] Rosenbaum PR Rubin DB Reducing bias in observational studies using subclassification on the propensity score J Am Stat Assoc 1984 16 516 524 10.1080/01621459.1984.10478078

[B10] Clec'h C Alberti C Vincent F Garrouste-Orgeas M de Lassence A Toledano D Azoulay E Adrie C Jamali S Zaccaria I Cohen Y Timsit JF Tracheostomy does not improve the outcome of patients requiring prolonged mechanical ventilation: a propensity analysis Crit Care Med 2007 16 132 138 10.1097/01.CCM.0000251134.96055.A6 17133180

[B11] Bellomo R Ronco C Kellum JA Mehta RL Palevsky P Acute renal failure - definition, outcome measures, animal models, fluid therapy and information technology needs: the Second International Consensus Conference of the Acute Dialysis Quality Initiative (ADQI) Group Crit Care 2004 16 R204 212 10.1186/cc2872 15312219 PMC522841

[B12] Joffe MM Rosenbaum PR Invited commentary: propensity scores Am J Epidemiol 1999 16 327 333 10.1093/oxfordjournals.aje.a010011 10453808

[B13] Brookhart MA Schneeweiss S Rothman KJ Glynn RJ Avorn J Sturmer T Variable selection for propensity score models Am J Epidemiol 2006 16 1149 1156 10.1093/aje/kwj149 16624967 PMC1513192

[B14] Rubin DB Thomas N Matching using estimated propensity scores: relating theory to practice Biometrics 1996 16 249 264 10.2307/2533160 8934595

[B15] Outcomerea http://outcomerea.org/Macros-SAS/Voir-categorie.html

[B16] Augustine JJ Sandy D Seifert TH Paganini EP A randomized controlled trial comparing intermittent with continuous dialysis in patients with ARF Am J Kidney Dis 2004 16 1000 1007 10.1053/j.ajkd.2004.08.022 15558520

[B17] Bellomo R Cass A Cole L Finfer S Gallagher M Lo S McArthur C McGuinness S Myburgh J Norton R Scheinkestel C Su S Intensity of continuous renal-replacement therapy in critically ill patients N Engl J Med 2009 16 1627 1638 19846848 10.1056/NEJMoa0902413

[B18] Mehta RL McDonald B Gabbai FB Pahl M Pascual MT Farkas A Kaplan RM A randomized clinical trial of continuous versus intermittent dialysis for acute renal failure Kidney Int 2001 16 1154 1163 10.1046/j.1523-1755.2001.0600031154.x 11532112

[B19] Palevsky PM Zhang JH O'Connor TZ Chertow GM Crowley ST Choudhury D Finkel K Kellum JA Paganini E Schein RM Smith MW Swanson KM Thompson BT Vijayan A Watnick S Star RA Peduzzi P Intensity of renal support in critically ill patients with acute kidney injury N Engl J Med 2008 16 7 20 18492867 10.1056/NEJMoa0802639 PMC2574780

[B20] Schiffl H Lang SM Fischer R Daily hemodialysis and the outcome of acute renal failure N Engl J Med 2002 16 305 310 10.1056/NEJMoa010877 11821506

[B21] Vinsonneau C Camus C Combes A Costa de Beauregard MA Klouche K Boulain T Pallot JL Chiche JD Taupin P Landais P Dhainaut JF Continuous venovenous haemodiafiltration versus intermittent haemodialysis for acute renal failure in patients with multiple-organ dysfunction syndrome: a multicentre randomised trial Lancet 2006 16 379 385 10.1016/S0140-6736(06)69111-3 16876666

[B22] Elseviers MM Lins RL Van der Niepen P Hoste E Malbrain ML Damas P Devriendt J SHARF investigators Renal replacement therapy is an independent risk factor for mortality in critically ill patients with acute kidney injury Crit Care 2010 16 R221 10.1186/cc9355 21122146 PMC3219996

[B23] Ahlstrom A Tallgren M Peltonen S Pettila V Evolution and predictive power of serum cystatin C in acute renal failure Clin Nephrol 2004 16 344 350 15571178 10.5414/cnp62344

[B24] Delanaye P Lambermont B Chapelle JP Gielen J Gerard P Rorive G Plasmatic cystatin C for the estimation of glomerular filtration rate in intensive care units Intensive Care Med 2004 16 980 983 10.1007/s00134-004-2189-5 14985953

[B25] Herget-Rosenthal S Marggraf G Husing J Goring F Pietruck F Janssen O Philipp T Kribben A Early detection of acute renal failure by serum cystatin C Kidney Int 2004 16 1115 1122 10.1111/j.1523-1755.2004.00861.x 15327406

[B26] Mishra J Dent C Tarabishi R Mitsnefes MM Ma Q Kelly C Ruff SM Zahedi K Shao M Bean J Mori K Barasch J Devarajan P Neutrophil gelatinase-associated lipocalin (NGAL) as a biomarker for acute renal injury after cardiac surgery Lancet 2005 16 1231 1238 10.1016/S0140-6736(05)74811-X 15811456

[B27] Nickolas TL O'Rourke MJ Yang J Sise ME Canetta PA Barasch N Buchen C Khan F Mori K Giglio J Devarajan P Barasch J Sensitivity and specificity of a single emergency department measurement of urinary neutrophil gelatinase-associated lipocalin for diagnosing acute kidney injury Ann Intern Med 2008 16 810 819 18519927 10.7326/0003-4819-148-11-200806030-00003 PMC2909852

[B28] Villa P Jimenez M Soriano MC Manzanares J Casasnovas P Serum cystatin C concentration as a marker of acute renal dysfunction in critically ill patients Crit Care 2005 16 R139 143 10.1186/cc3044 15774046 PMC1175926

[B29] Wagener G Jan M Kim M Mori K Barasch JM Sladen RN Lee HT Association between increases in urinary neutrophil gelatinase-associated lipocalin and acute renal dysfunction after adult cardiac surgery Anesthesiology 2006 16 485 491 10.1097/00000542-200609000-00011 16931980

[B30] Zappitelli M Washburn KK Arikan AA Loftis L Ma Q Devarajan P Parikh CR Goldstein SL Urine neutrophil gelatinase-associated lipocalin is an early marker of acute kidney injury in critically ill children: a prospective cohort study Crit Care 2007 16 R84 10.1186/cc6089 17678545 PMC2206519

[B31] Ostermann M Chang RW Acute kidney injury in the intensive care unit according to RIFLE Crit Care Med 2007 16 1837 1843 quiz 1852 10.1097/01.CCM.0000277041.13090.0A 17581483

[B32] Uchino S Bellomo R Goldsmith D Bates S Ronco C An assessment of the RIFLE criteria for acute renal failure in hospitalized patients Crit Care Med 2006 16 1913 1917 10.1097/01.CCM.0000224227.70642.4F 16715038

[B33] Hoste EA Clermont G Kersten A Venkataraman R Angus DC De Bacquer D Kellum JA RIFLE criteria for acute kidney injury are associated with hospital mortality in critically ill patients: a cohort analysis Crit Care 2006 16 R73 10.1186/cc4915 16696865 PMC1550961

[B34] Hoste EA Kellum JA Acute kidney injury: epidemiology and diagnostic criteria Curr Opin Crit Care 2006 16 531 537 10.1097/MCC.0b013e3280102af7 17077682

