# Supplementary material for: Efficacy of renal replacement therapy in critically ill patients: a propensity analysis
Source: Crit Care. 2012 Dec 19;16(6):R236. doi: 10.1186/cc11905 (PMC3672625; doi:10.1186/cc11905)
Supplement: Additional file 1 — Baseline characteristics of RIFLE R class patients with and without renal replacement therapy (RRT). [file cc11905-S1.DOC]

**Additional file 1. Baseline characteristics of RIFLE R class patients with and without renal replacement therapy (RRT).**

| Variable | Patients with RRT  (n = 41) | Patients without RRT  (n = 984) | *P* value |
| --- | --- | --- | --- |
| Age, mean (SD) | 59.2 (17.0) | 67.9 (15.6) | < 0.001 |
| Males, no. (%) | 32 (78.1) | 556 (56.5) | 0.006 |
| SAPS II score, mean (SD) | 57.6 (20.4) | 44.7 (17.1) | < 0.0001 |
| APACHE II score, mean (SD) | 22.3 (6.9) | 17.8 (6.5) | < 0.0001 |
| Transfer from ward, no. (%) | 19 (46.3) | 458 (46.6) | 0.98 |
| McCabe, no. (%) | | | |
| 1 | 17 (41.5) | 591 (60.1) | 0.03 |
| 2 | 18 (43.9) | 324 (32.9) |
| 3 | 6 (14.6) | 69 (7.0) |
| Admission category, no. (%) | | | |
| Medical | 32 (78.1) | 722 (73.4) | 0.79 |
| Scheduled surgery | 4 (9.8) | 126 (12.8) |
| Unscheduled surgery | 5 (12.2) | 136 (13.8) |
| Chronic coexisting conditions, no. (%) | | | |
| Cardiac disease | 11 (26.8) | 174 (17.7) | 0.14 |
| Respiratory disease | 7 (17.1) | 158 (16.1) | 0.86 |
| Liver disease | 5 (12.2) | 56 (5.7) | 0.09 |
| Immunodeficiency | 8 (19.5) | 135 (13.7) | 0.29 |
| Uncomplicated diabetes mellitus | 8 (19.5) | 117 (11.9) | 0.15 |
| Complicated diabetes mellitus | 0 (0) | 45 (4.6) | 0.25 |

SAPS, Simplified Acute Physiology Score; APACHE, Acute Physiology and Chronic Health Evaluation.
