# Supplementary material for: Efficacy of renal replacement therapy in critically ill patients: a propensity analysis
Source: Crit Care. 2012 Dec 19;16(6):R236. doi: 10.1186/cc11905 (PMC3672625; doi:10.1186/cc11905)
Supplement: Additional file 2 — Baseline characteristics of RIFLE I class patients with and without renal replacement therapy (RRT). [file cc11905-S2.DOC]

**Additional file 2. Baseline characteristics of RIFLE I class patients with and without renal replacement therapy (RRT).**

| Variable | Patients with RRT  (n = 110) | Patients without RRT  (n = 720) | *P* value |
| --- | --- | --- | --- |
| Age, mean (SD) | 61.6 (17.0) | 67.5 (15.3) | < 0.001 |
| Males, no. (%) | 74 (67.3) | 428 (59.4) | 0.12 |
| SAPS II score, mean (SD) | 61.9 (21.2) | 50.4 (20.8) | < 0.0001 |
| APACHE II score, mean (SD) | 21.8 (6.9) | 20.5 (7.2) | 0.07 |
| Transfer from ward, no. (%) | 58 (52.7) | 329 (45.7) | 0.17 |
| McCabe, no. (%) | | | |
| 1 | 60 (54.6) | 416 (57.8) | 0.75 |
| 2 | 42 (38.2) | 248 (34.4) |
| 3 | 8 (7.2) | 56 (7.8) |
| Admission category, no. (%) | | | |
| Medical | 85 (77.3) | 507 (70.4) | 0.10 |
| Scheduled surgery | 5 (4.5) | 80 (11.1) |
| Unscheduled surgery | 20 (18.2) | 133 (18.5) |
| Chronic coexisting conditions, no. (%) | | | |
| Cardiac disease | 16 (14.6) | 147 (20.4) | 0.15 |
| Respiratory disease | 10 (9.1) | 91 (12.6) | 0.29 |
| Liver disease | 16 (14.6) | 43 (6.0) | 0.004 |
| Immunodeficiency | 28 (25.5) | 109 (15.7) | 0.09 |
| Uncomplicated diabetes mellitus | 10 (9.1) | 80 (11.1) | 0.52 |
| Complicated diabetes mellitus | 3 (2.7) | 37 (5.1) | 0.27 |

SAPS, Simplified Acute Physiology Score; APACHE, Acute Physiology and Chronic Health Evaluation.
