# Supplementary material for: Efficacy of renal replacement therapy in critically ill patients: a propensity analysis
Source: Crit Care. 2012 Dec 19;16(6):R236. doi: 10.1186/cc11905 (PMC3672625; doi:10.1186/cc11905)
Supplement: Additional file 3 — Baseline characteristics of RIFLE F class patients with and without renal replacement therapy (RRT). [file cc11905-S3.DOC]

**Additional file 3. Baseline characteristics of RIFLE F class patients with and without renal replacement therapy (RRT).**

| Variable | Patients with RRT  (n = 394) | Patients without RRT  (n = 597) | *P* value |
| --- | --- | --- | --- |
| Age, mean (SD) | 61.4 (16.4) | 67.3 (15.4) | < 0.0001 |
| Males, no. (%) | 257 (65.2) | 325 (54.4) | < 0.001 |
| SAPS II score, mean (SD) | 55.3 (18.2) | 52.8 (21.6) | 0.05 |
| APACHE II score, mean (SD) | 21.3 (7.0) | 21.5 (7.2) | 0.53 |
| Transfer from ward, no. (%) | 214 (54.3) | 285 (47.7) | 0.04 |
| McCabe, no. (%) | | | |
| 1 | 237 (60.2) | 345 (57.8) | 0.53 |
| 2 | 128 (32.5) | 199 (33.3) |
| 3 | 29 (7.3) | 53 (8.9) |
| Admission category, no. (%) | | | |
| Medical | 271 (68.8) | 426 (71.3) | 0.63 |
| Scheduled surgery | 43 (10.9) | 53 (8.9) |
| Unscheduled surgery | 80 (20.3) | 118 (19.8) |
| Chronic coexisting conditions, no. (%) | | | |
| Cardiac disease | 62 (15.7) | 99 (16.6) | 0.72 |
| Respiratory disease | 38 (9.6) | 62 (10.4) | 0.71 |
| Liver disease | 29 (7.4) | 29 (4.9) | 0.004 |
| Immunodeficiency | 28 (25.5) | 109 (15.7) | 0.11 |
| Uncomplicated diabetes mellitus | 45 (11.4) | 60 (10.1) | 0.49 |
| Complicated diabetes mellitus | 27 (6.9) | 36 (6.0) | 0.61 |

SAPS, Simplified Acute Physiology Score; APACHE, Acute Physiology and Chronic Health Evaluation.
