# Supplementary material for: Efficacy of renal replacement therapy in critically ill patients: a propensity analysis
Source: Crit Care. 2012 Dec 19;16(6):R236. doi: 10.1186/cc11905 (PMC3672625; doi:10.1186/cc11905)
Supplement: Additional file 4 — Lengths of ICU stay after reaching maximum RIFLE class in patients with and without renal replacement therapy (RRT). [file cc11905-S4.DOC]

**Additional file 4. Lengths of intensive care unit stay after reaching maximum RIFLE class in patients with and without renal replacement therapy (RRT).**

|  | Patients with RRT | Patients without RRT | *P* value |
| --- | --- | --- | --- |
| *All patients* | *N = 545* | *N = 2301* |  |
| Days, median [interquartile range] | 13 [7-26] | 6 [3-11] | < 0.0001 |
| *R class patients* | *N = 41* | *N = 984* |  |
| Days, median [interquartile range] | 12 [4-18] | 5 [3-10] | 0.001 |
| *I class patients* | *N = 110* | *N = 720* |  |
| Days, median [interquartile range] | 11 [6-22] | 6 [3-11] | < 0.0001 |
| *F class patients* | *N = 394* | *N = 597* |  |
| Days, median [interquartile range] | 14 [7-27] | 6 [3-11] | < 0.0001 |
