# Supplementary material for: Efficacy of renal replacement therapy in critically ill patients: a propensity analysis
Source: Crit Care. 2012 Dec 19;16(6):R236. doi: 10.1186/cc11905 (PMC3672625; doi:10.1186/cc11905)
Supplement: Additional file 5 — Lengths of ICU stay after reaching maximum RIFLE class in nonsurvivors with and without renal replacement therapy (RRT). [file cc11905-S5.DOC]

**Additional file 5. Lengths of intensive care unit stay after reaching maximum RIFLE class in non survivors with and without renal replacement therapy (RRT).**

|  | Patients with RRT | Patients without RRT | *P* value |
| --- | --- | --- | --- |
| *All patients* | *N = 207* | *N = 403* |  |
| Days, median [interquartile range] | 10 [5-19] | 3 [2-8] | < 0.0001 |
| *R class patients* | *N = 19* | *N = 101* |  |
| Days, median [interquartile range] | 9 [3-16] | 4 [2-10] | 0.02 |
| *I class patients* | *N = 59* | *N = 170* |  |
| Days, median [interquartile range] | 9 [4-21] | 3 [2-8] | < 0.0001 |
| *F class patients* | *N = 129* | *N = 132* |  |
| Days, median [interquartile range] | 11 [6-19] | 3 [2-7] | < 0.0001 |
