# Supplementary material for: Efficacy of renal replacement therapy in critically ill patients: a propensity analysis
Source: Crit Care. 2012 Dec 19;16(6):R236. doi: 10.1186/cc11905 (PMC3672625; doi:10.1186/cc11905)
Supplement: Additional file 6 — Association of renal replacement therapy (RRT) with hospital mortality in multivariate conditional logistic regression according to timing of RRT and maximum RIFLE class reached during the ICU stay (model 1). [file cc11905-S6.DOC]

**Additional file 6. Association of renal replacement therapy (RRT) with hospital mortality in multivariate conditional logistic regression according to timing of RRT and maximum RIFLE class reached during the ICU stay (model 1).**

|  | OR | 95% CI | *P* value |
| --- | --- | --- | --- |
| **Immediate RRT*** |  |  |  |
| R class patients^┼^ | - | - | - |
| I class patients | 1.77 | 0.44-7.15 | 0.42 |
| F class patients | 1.31 | 0.64-2.67 | 0.62 |
| **Early RRT**** |  |  |  |
| R class patients^┼^ | - | - | - |
| I class patients^┼^ | - | - | - |
| F class patients | 0.83 | 0.27-2.57 | 0.74 |
| **Delayed RRT***** |  |  |  |
| R class patients^┼^ | - | - | - |
| I class patients^┼^ | - | - | - |
| F class patients | 2.59 | 0.78-8.62 | 0.12 |

OR, odds ratio; CI, confidence interval.

* initiated within 24 hrs after reaching maximum RIFLE class.

** initiated between 24 and 48 hrs after reaching maximum RIFLE class.

*** initiated more than 48 hrs after reaching maximum RIFLE class.

^┼^ No analysis performed due to the small sample size.
