# Supplementary material for: Efficacy of renal replacement therapy in critically ill patients: a propensity analysis
Source: Crit Care. 2012 Dec 19;16(6):R236. doi: 10.1186/cc11905 (PMC3672625; doi:10.1186/cc11905)
Supplement: Additional file 8 — Association of renal replacement therapy (RRT) with hospital mortality in multivariate conditional logistic regression (matched patients) according to timing of RRT: results of sensitivity analyses including only patients with a normal serum creatinine value measured on ICU admission. [file cc11905-S8.DOC]

**Additional file 8. Association of renal replacement therapy (RRT) with hospital mortality in multivariate conditional logistic regression (matched patients) according to timing of RRT: results of sensitivity analyses including only patients with a normal serum creatinine value measured on ICU admission.**

|  | OR | 95% CI | *P* value |
| --- | --- | --- | --- |
| **Model 1** |  |  |  |
| All RRT (whatever the timing) | 1.73 | 0.70-4.27 | 0.24 |
| Immediate RRT* | 1.58 | 0.58-4.26 | 0.37 |
| Early RRT** | 1.15 | 0.21-3.13 | 0.87 |
| Delayed RRT*** | 3.91 | 1.35-11.30 | 0.01 |
| **Model 2** |  |  |  |
| All RRT (whatever the timing) | 2.87 | 0.84-9.78 | 0.09 |
| Immediate RRT* | 1.54 | 0.54-4.24 | 0.41 |
| Early RRT** | 1.26 | 0.12-13.10 | 0.84 |
| Delayed RRT*** | 2.88 | 1.12-7.42 | 0.03 |

OR, odds ratio; CI, confidence interval.

* initiated within 24 hrs after reaching maximum RIFLE class.

** initiated between 24 and 48 hrs after reaching maximum RIFLE class.

*** initiated more than 48 hrs after reaching maximum RIFLE class.
